# A dual-transmission-bands rasorber with improved absorption and oblique incidence performance

**DOI:** 10.1038/s41598-022-25074-9

**Published:** 2022-11-29

**Authors:** Zheyipei Ma, Chao Jiang, Jiale Li, Xiaozhong Huang

**Affiliations:** Powder Metallurgy Research Institute of Central South University, Changsha, 410083 China

**Keywords:** Metamaterials, Metamaterials, Electronic properties and materials

## Abstract

In this paper, a high-absorption and dual-transmission-bands rasorber (HADTR) was proposed. Different from the reported designs, a foam layer and a FR4 layer are added as top layers in HADTR to improve its absorption and oblique incidence performance. The unit cell of resistive layer is concentric metallic rings loaded with chip resistors based on absorption enforced design to ensure its dual-transmission-bands at the same time. The unit cell of band-stop frequency selective surface (BS-FSS) is double metallic square loops loaded with chip inductors on both sides of FR4 substrate, which expands its reflection band (|S_11_|≥ − 1.0 dB) without destroying its dual-transmission-bands effectively. At normal incidence, for TE and TM polarization, the HADTR has a low-frequency passband up to 1.34 GHz (|S_21_|≥ − 1.5 dB), a high-frequency passband from 16.04 to 18.00 GHz (|S_21_|≥ − 1.5 dB) and a wide 90% absorption band from 5.01 to 10.56 GHz; and the reflection coefficient below − 10 dB and − 20 dB is in the range of 4.48–11.70 GHz and 5.48–9.96 GHz, respectively. For TE/TM polarization, the 90% absorption of oblique incidence stability is 40° and 30° respectively. Strong association between measurement and simulation results validates the design method and the HADTR.

## Introduction

Frequency selective rasorbers^1,2^ (FSRs) have been widely studied due to their potential in the fields of electromagnetic wave applications like Radar cross-section reduction, electromagnetic compatibility and radomes, etc. The conventional metallic frequency selective surface (FSS) is transparent to incident electromagnetic waves in certain passbands and reflects them outside of the passbands. Different from FSS, the FSRs absorbs the out-band signals rather than reflecting them to reduce not only monostatic radar cross section (RCS) but also bistatic RCS. According to the geometric structures of FSRs, they can be divided into two categories: two-dimensional (2-D) FSRs^3–5^ and three-dimensional (3-D) FSRs^6–8^. 2-D FSRs consist of a lossless metallic FSS for passbands and one or more lossy layers for absorption bands. 3-D FSRs usually contain energy consumption parts, such as chip resistors^9,10^, magnetic loss materials structures^11,12^, resistive sheets^12,13,17^, and lossless resonators constructed. Limited by the intricate structure and high costs of 3-D FSRs, 2-D FSRs with high absorption bands and low insertion loss pass bands are becoming excellent candidates for research, design, and applications.

In previous studies, most of 2-D FSRs are constructed by cascading lossy layers above the metallic FSS layers. The lossy layer generally has wide absorption bands out of the transmission bands. The metallic FSS layer should have the same transmission bands with that of the lossy layer to ensure low insertion loss and high reflection bands corresponding with absorption bands of the lossy layer to strengthen the absorptivity and broaden the absorption bandwidth of FSRs. Based on the relative positions of passbands and absorption bands, 2-D FSRs in the past literatures can be sorted into four types, which are respectively: (1) low-frequency absorption and high-frequency transmission (AT)^14,16,17^; (2) low-frequency transmission and high-frequency absorption (TA)^13,18–20^; (3) low-frequency absorption, intermediate-frequency transmission and high-frequency absorption (ATA)^15,21–25^; (4) low-frequency transmission, intermediate-frequency absorption and high-frequency transmission (TAT)^26–31^.

Most of reported FSRs either focus on improving the absorbing performance of the absorbing band or reducing the insertion loss of the transmitting band. There are few studies that take into account the absorption performance, transmission characteristics and oblique incidence stability at once. In fact, the absorption performance, transmission characteristics and oblique incidence stability must be considered together, if FSRs were widely applied in communications and radar systems with multiple operating frequency bands. Absorption bands of existing FSRs are usually measured by − 10 dB reflection^17,24,26,30^, which cannot precisely represent 90% absorption the same as absorber. For example, although a single layer resistive FSS can also produce a wide − 10 dB reflection band and low insertion loss transmission bands, this − 10 dB reflection band will not be regarded as an effective absorption band.

In this work, a possible method is proposed to design low insertion loss transmission and high absorption TAT FSR. In order to achieve high absorptivity, a new two-steps design method is proposed; firstly, composition element by absorption enforced design is used as unit cell for resistive layer initially; then PMI and FR4 superstrate is added on the top of resistive layer to improve absorption and oblique incidence stability further. To design a BS-FSS with a high reflection band and dual-transmission bands for FSRs, a modified method for synthesizing wideband reflection and high transmission BS-FSSs is demonstrated, which consists of chip inductors loaded metal FSS design and impedance match layer; and in this paper, the unit cell of BS-FSS is chip inductors loaded double metallic square loops on both sides of FR4 substrate. The proposed HADTR has a low-frequency passband up to 1.34 GHz (|S_21_|≥ − 1.5 dB), a high-frequency passband from 16.04 to 18.00 GHz (|S_21_|≥ − 1.5 dB) and a wide 90% absorption band from 5.01 to 10.56 GHz; and the reflection coefficient below − 10 dB and − 20 dB is in the range of 4.48–11.70 GHz and 5.48–9.96 GHz, respectively. And under the 40° oblique incidence, the HADTR can still maintain the absorptivity higher than 90% from 5.52 to 10.68 GHz. Finally, the design was fabricated, and the measurement results are compared with the simulation results to corroborate the proposed method.

## Results

### Design principles and simulation results

**Figure 1:**
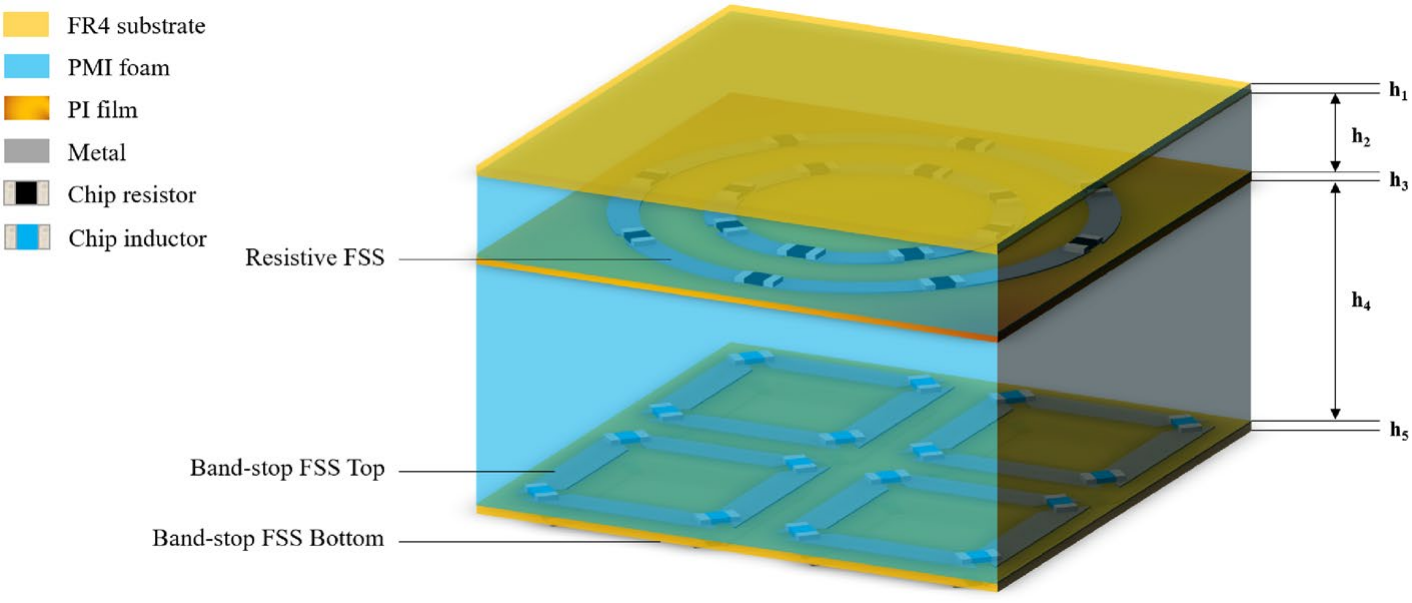
Geometrical configuration of the proposed HADTR.

Existing FSRs were usually designed and analyzed by Smith chart^2^, wave-splitting method^18^, equivalent circuit model method^13–17,19–22,24–31^ and characteristic modes analysis^23^. In this paper, the proposed HADTR is designed from two parts. The first part is rapid design of a high-absorption Circuit-Analog (CA) absorber based on the characteristics of Salisbury screen and Snell’s law. The second part is design of a frequency selective surface (BS-FSS). Figure 1 shows the 3-D topology of the proposed HADTR, which consist of a BS-FSS, a foam layer, a CA sheet, a foam layer and a FR4 layer.

**Figure 2:**
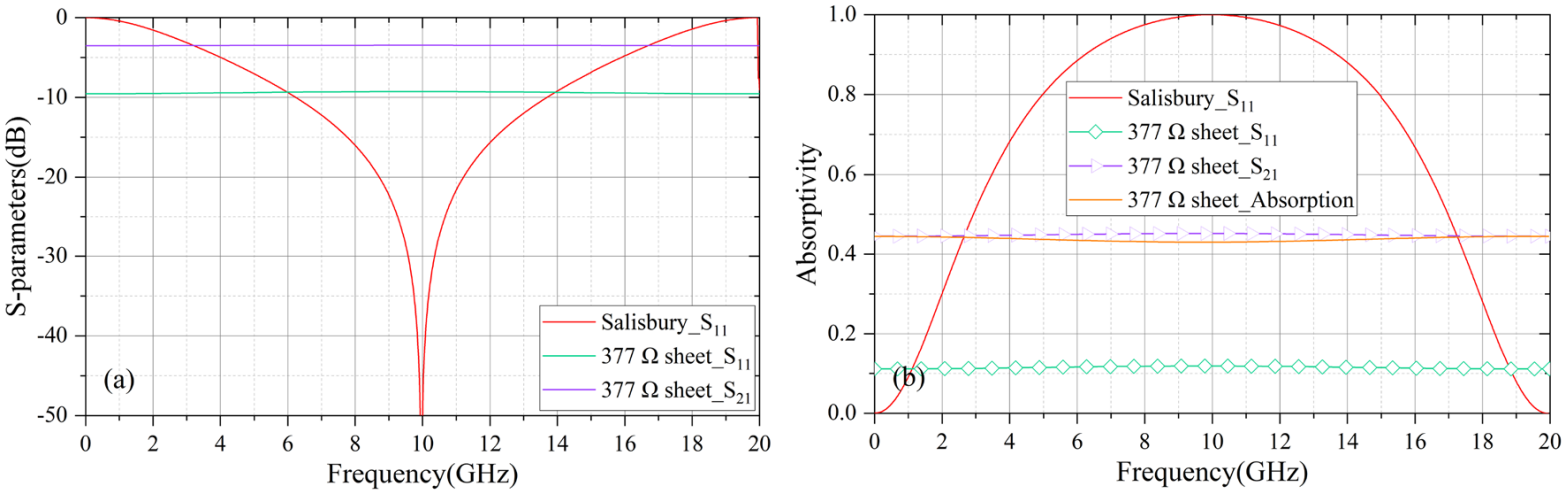
At normal incidence, under TE polarization. (**a**) reflection coefficient of a Salisbury Screen with ε_0_ = 1.0, d_1_ = 7.50 mm, and the reflection/transmission coefficient of a 377 Ω/sq sheet; (**b**) the absorptivity of the Salisbury Screen and the reflectance, transmittance, and absorptivity of the 377 Ω/sq sheet. (R =|S_11_|^2^, T =| S_21_|^2^, A = 1 −|S_21_|^2^ −|S_11_|^2^).

The CA absorber for HADTR includes a CA sheet and three dielectric layers. The CA absorber derives from Salisbury Screen where impedance of resistive layer is equivalent to wave impedance of free space Z_0_ (377 Ω/sq), so its frequency response characteristics can also be used in the design of CA sheet. By simulation software CST, we quantified the relationship among reflectance, transmittance, and absorptivity of the resistive layer to clarify the function of the resistive sheet in the Salisbury Screen furtherly. At normal incidence, Fig. 2a illustrates the reflection coefficient of a Salisbury Screen with ε_0_ = 1.0, h_1_ = 7.50 mm, and the reflection/transmission coefficient of a 377 Ω/sq sheet; and − 20 dB absorption band of Salisbury Screen is from 8.74 to 11.26 GHz. Figure 2b shows the absorptivity of the Salisbury Screen and the reflectance, transmittance, and absorptivity of the 377 Ω/sq sheet. The relationship of absorptivity A, reflectance |S_11_|^2^ and transmittance |S_21_|^2^ is

1 $$A = \, 1 \, - \, \left| {S_{21} } \right|^{2} - \left| {S_{11} } \right|^{2}$$

where |S_11_| is reflection coefficient, and |S_21_| is transmission coefficient.

From 0 to 20 GHz, it can be observed that the energy E of normal incident plane wave will be divided into E_1_, E_2_ and E_3_ by 377 Ω/square resistive sheet, which are about 44.5%, 11.0% and 44.5% of E respectively; the corresponding transmission coefficient |S_21_| is about − 3.5 dB, and the reflection coefficient |S_11_| is about − 9.5 dB. Comparing Fig. 2a with b, it can be concluded that Salisbury Screen cannot maximize the electrical dissipation in every frequency band, which is caused by a corresponding relationship between resonant frequency and distance h_1_. However, under the ideal impedance matching, Salisbury Screen still has a − 20 dB absorption band from 8.74 to 11.26 GHz rather than an absorption frequency point, which illustrates that the CA sheet with 44% energy dissipation of the incident energy is an important factor for high-absorption absorber. Besides, the transmission energy E_3_ should be greater than the reflected energy E_2_ as much as possible to achieve the optimal interference consumption furtherly. The thickness of different dielectric layers will be designed according to the mechanisms of Salisbury Screen. In addition, FR4 slab h_1_ and PMI slab h_2_ are significant for high-absorption properties of a CA absorber^32^.

**Figure 3:**
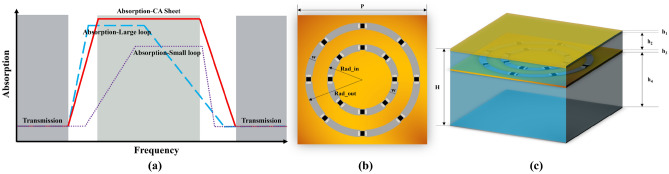
Geometrical configuration of the proposed CA absorber. (**a**) Design goal of unit cell; (**b**) Unit cell of the CA sheet; (**c**) Perspective view of proposed absorber.

**Figure 4:**
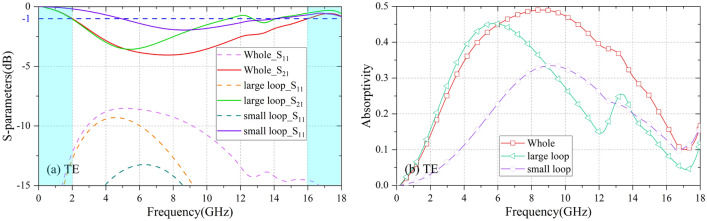
At normal incidence, frequency response and absorptivity of concentric rings, single large resistive loop, and single small resistive loop CA sheets. Reflection coefficient R/Transmission coefficient T/Absorptivity A of proposed CA sheet (R =|S_11_|, T =|S_21_|, A = 1 −|S_21_|^2^ −|S_11_|^2^). P = 22.0 mm, Rad_out = 9.0 mm, Rad_in = 6.0 mm, w = 0.5 mm, h_3_ = 0.13 mm, R_out_ = 140 Ω, R_in_ = 120 Ω.

As shown in Fig. 3a and b, different from the existing designs that adopt composition units to expand absorption bands, the novelty of proposed CA sheet is its absorption enforced unit cell, which consists of a large resistive loop for low frequency absorption and a small resistive loop for absorption compensation of high frequency. It should be noticed that this method can design high absorption absorber with simple geometric units, which can improve efficiency of whole structure design. CA sheet is printed on the PI film; both large and small rings are loaded with eight chip resistors. The parameters are P = 22.0 mm, Rad_out = 9.0 mm, Rad_in = 6.0 mm, w = 0.50 mm, h_3_ = 0.13 mm. Figure 4 illustrates the frequency response and absorptivity of concentric rings, single large resistive loop and small loop CA sheets at normal incidence. The single large resistive loop CA sheet has an absorption peak (A = 0.455) at 5.78 GHz; the transparent windows (|S_21_|≥ − 1.0 dB) are in the range of 0–1.99 GHz and 14.03–18.43 GHz, respectively. The single small resistive loop CA sheet has an absorption peak (A = 0.336) at 9.12 GHz; the transmission bands (|S_21_|≥ − 1.0 dB) are in the range of 0 to 4.91 GHz and 14.52 to 18.22 GHz, respectively. As a result, the proposed CA sheet can absorb at least 44% and 40% energy of incident plane wave energy in the range of 5.69 to 11.04 GHz and 4.78 to 11.88 GHz, respectively. And the transmission bands (|S_21_|≥ − 1.0 dB) of the proposed CA sheet are in the range of 0–2.02 GHz and 16.04–18.40 GHz, respectively.

Figure 3c shows the whole structure of proposed CA absorber, which consist of a metallic ground, a foam layer, a CA sheet, a foam layer and a FR4 layer. The hard protective FR4 substrate of h_1_ can improve absorption performance and longevity of CA absorber; the PMI layer serves as support layer for FR4, h_1_. The thickness of different dielectric layers can be obtained by quarter wavelengths of f_5.78 GHz_ and f_9.12 GHz_. To enforce the absorption peaks at 5.78 GHz (A = 0.455) and 9.12 GHz (A = 0.336), The whole thickness H and the value h_4_ are calculated by these two absorption peaks. Therefore, the value of whole thickness H corresponds to λ _(5.78 GHz)_/4; and the value of h_4_ interrelates with λ _(9.12 GHz)_/4.

**Figure 5:**
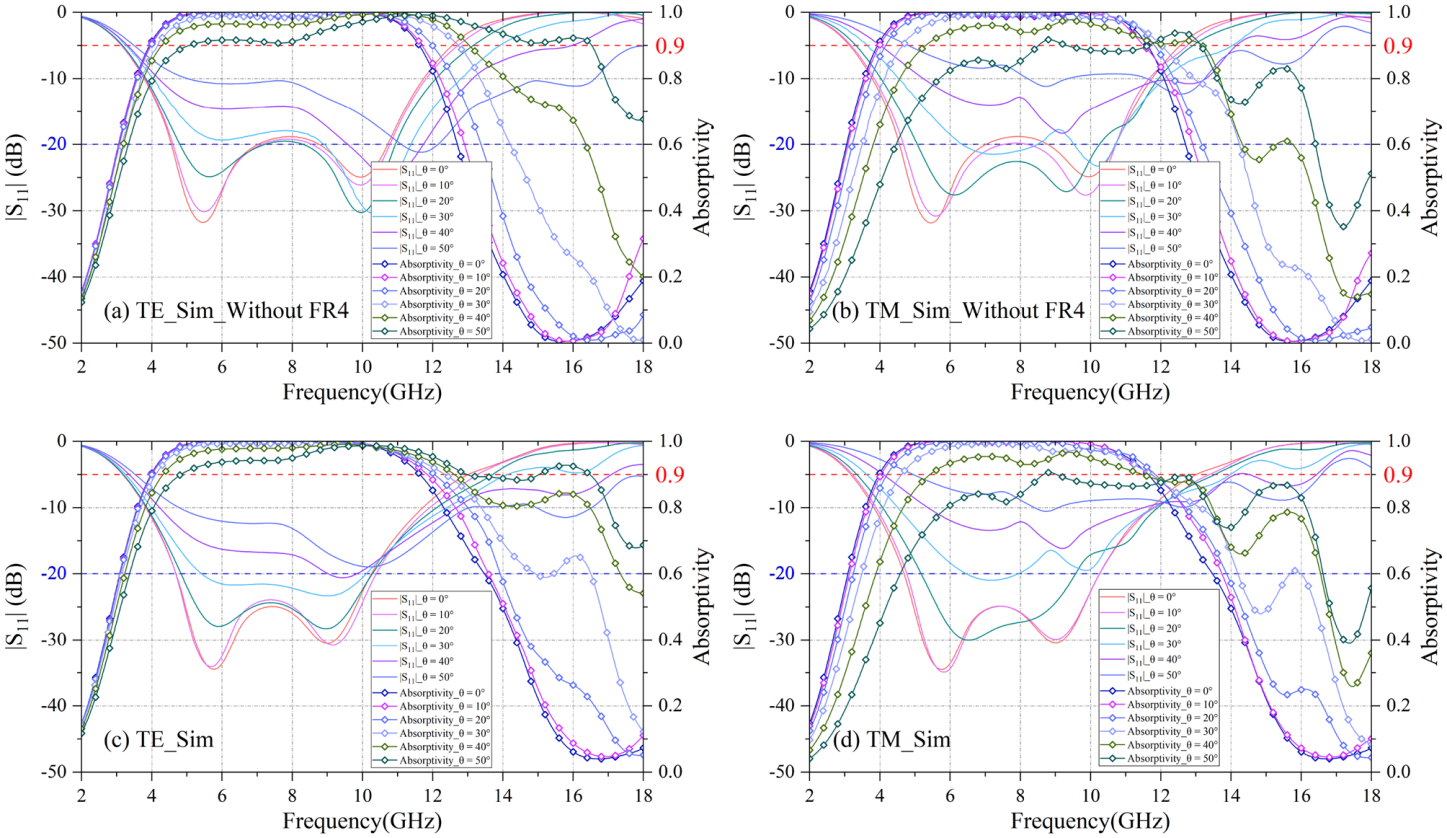
Simulation results of reflection coefficients and absorptivity under different oblique incidences of the proposed CA absorber with/without FR4 superstrate. P = 22.0 mm, Rad_out = 9.0 mm, Rad_in = 6.0 mm, w = 0.5 mm, h_1_ = 0.30 mm, h_2_ = 2.50 mm, h_3_ = 0.13 mm, h_4_ = 8.50 mm, R_out_ = 140 Ω, R_in_ = 120 Ω. (**a**) TE-polarization without FR4 superstrate; (**b**) TM-polarization without FR4 superstrate; (**c**) TE-polarization with FR4 superstrate; (**d**) TM-polarization with FR4 superstrate.

The wavelength λ can be calculated by *λ*_*0*_ = *c*_*0*_*/f, λ*_*m*_ = *λ*_*0*_*/*$\sqrt{{\varepsilon }_{r}{\mu }_{r}}$, where c_0_ (2.99 × 10^8^ m/s) is velocity of light; f is frequency point; λ_0_ is wavelength corresponding to the f; λ_m_ is the wavelength in dielectric materials. If all the dielectric layers are air, the value of H should satisfy H_air_ = λ _(5.78 GHz)_/4 = 12.93 mm, h_4_ = λ _(9.12 GHz)_/4 = 8.20 mm. On the one hand, in practice, the dielectric layer h_1_ is FR4 with ε_r_ of 4.4; dielectric slabs h_2_ and h_4_ are PMI foam with ε_r_ of 1.05, that all the ε_r_ is greater than the permittivity of air; and the substrate h_3_ of CA sheet is PI film with ε_r_ of 3.5; on the other hand, the values of h_1_ and h_3_ are much smaller than the value of h_2_ and h_4_. Therefore, assuming that all the dielectric layers of the structure are PMI foam, the value of H should satisfy H_PMI_ ~ λ _(5.78 GHz)_/4 = 12.63 mm; the value of h_4_ should meet h_4_PMI_ ~ λ _(9.12 GHz)_/4 = 8.00 mm. Considering the usual thickness of FR4 substrate and PI film, we assigned the initial values h_1_FR4_ = 0.3 mm, h_3_PI_ = 0.13 mm, which equivalent value in PMI foam are h_1_PMI_ = 0.63 mm, h_3_PMI_ = 0.24 mm. So that the thickness value of h_2_ ~ H_PMI_ − h_1_PMI_ − h_3_PMI_ − h_4_PMI_ = 3.76 mm. After that, we use the full-wave simulation software CST to furtherly optimize the parameters. And the simulation results of the final design are demonstrated in Fig. 5; and the parameters are P = 22.0 mm, Rad_out = 9.0 mm, Rad_in = 6.0 mm, w = 0.5 mm, h_1_ = 0.30 mm, h_2_ = 2.50 mm, h_3_ = 0.13 mm, h_4_ = 8.50 mm, R_out_ = 140 Ω, R_in_ = 120 Ω.

Simulation results of reflection coefficients and absorptivity under different oblique incidences of the proposed CA absorber without FR4 superstrate are shown in Fig. 5a,b. It should be noticed that the proposed concentric rings unit has absorption enforced property clearly; in the range of 4.56 – 10.70 GHz, the reflection coefficient of absorber without top FR4 layer h_1_ is very close to − 20 dB. After the FR4 superstrate is added, the absorption performance is improved furtherly. As shown in Fig. 5c,d, at normal oblique incidence, both TE and TM polarization simulation results demonstrate that the reflection coefficient of the CA absorber below − 10 dB is from 3.88 to 11.70 GHz (fractional bandwidth (FBW) = 101.2%); and the reflection coefficient below − 20 dB covers a bandwidth from 4.76 to 10.20 GHz (FBW = 72.7%). Under TE polarization, the proposed CA absorber always maintains an absorption bandwidth greater than 7.82 GHz (FBW ≥ 96.6%) with − 10 dB absorption, when the incident angle is less than 50°; and within the incident angle of 30°, the bandwidth of − 20 dB absorption is larger than 4.78 GHz (FBW ≥ 62.0%). Under TM polarization, the proposed CA absorber always maintains an absorption bandwidth greater than 6.22 GHz (FBW ≥ 76.6%) with − 10 dB absorption, when the incident angle is less than 30°.

**Figure 6:**
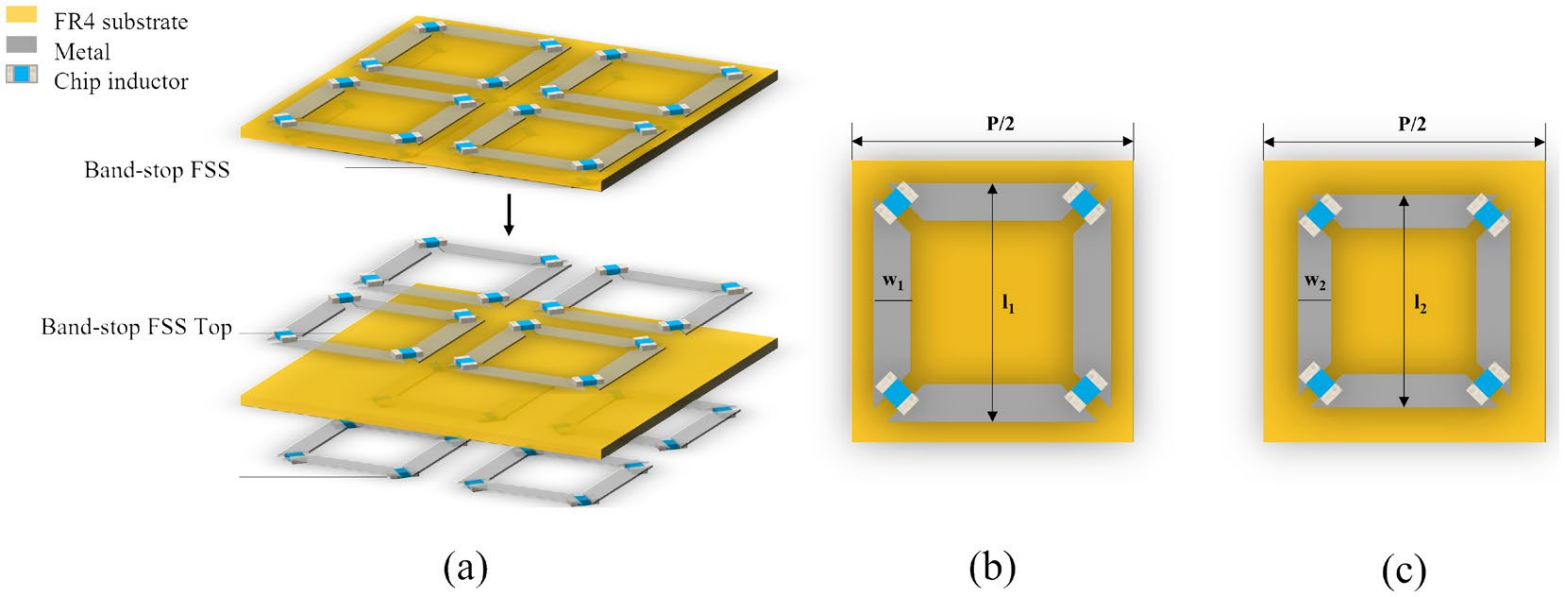
Geometrical configuration of the proposed BS-FSS. P = 22.0 mm, *l*_1_ = 10.4 mm, w_1_ = 1.7 mm, *l*_2_ = 9.0 mm, w_2_ = 1.1 mm, L_1_ = 0.4 nH, L_2_ = 0.1 nH. (**a**) Perspective view. (**b**) Unit cell of the top side band stop FSS. (**c**) Unit cell of the bottom side band stop FSS.

The common method to broaden reflection bandwidth is using double layers BS-FSS design^33^ with tradeoff of low transmission coefficient at transparent windows; however, high transmission coefficients are also important for FSRs design. In this part, a modified method for synthesizing wideband reflection and high transmission BS-FSSs is demonstrated, which consists of chip inductors loaded metal FSS design and impedance match layer. Though, the FSS with metallic loop unit cell is a kind of typical BS-FSS which can provide a narrow reflection band and two transmission bands, wide − 1 dB refection band^18^ of a BS-FSS is needed for wide 90% absorption band of an FSR. To ensure the BS-FSS with two wide transmission bands and a wide reflection band, we combined two distinct FSSs based on lumped-inductor-loaded large/small square metallic loops on both sides of a FR4 substrate with the same periodicity. A wide reflection band is obtained by superimposing of two narrow reflection bands, while the bandwidth of the transmission bands on both sides are not affected. As shown in Fig. 6, the top side BS-FSS determines the upper limit of the transmission band at low frequency and lower limit of the intermediate-frequency reflection band; the bottom side BS-FSS decides the starting frequency point of the transmission band at high frequency and the ending frequency point of the intermediate-frequency reflection band. Then, top layer FR4 slab also plays a role of impedance matching layer for transmission window^1^, which can obviously improve the transmission performance at high frequency.

References^33^ and^34^ analyzed arrays of single layer square loops and dual-layer square loops FSS structure respectively by equivalent circuit model. In Ref.^33^, the array of single layer square loops has a resonant frequency given by

2 $${f}_{0}=\frac{1}{2\pi \sqrt{{L}_{0}{C}_{0}}}$$

In Ref.^34^, the reactance L_0_ and susceptance C_0_ is given by

3 $$\frac{{L}_{0}}{{Z}_{0}}=\frac{l}{p}F\left(p, 2w, \lambda \right)$$

4 $$\frac{{C}_{0}}{{Z}_{0}}=4\frac{l}{p}F\left(p, g, \lambda \right)$$

where Z_0_ is the characteristic impedance of free space; p is the period of the array; *l* is the length of the square loop; w is the width of the loop sides; λ is the wavelength of plane wave; g is the gap between the loop sides.

In Ref.^14^, the researchers inserted with a circular spiral resonator (CSR), which was equal to a large inductor, in the center of the each of hexagonal metallic loop (HML) side; the diameter of the CSR is about 1.6 mm. Comparing the reflection/transmission coefficients of a single HML and the HML loaded with CSRs, the added CSRs shifted the resonance frequency and high frequency transparent window of HML to lower frequency; at the same time, the inserted CSRs decreases the reflection bandwidth of HML. However, the size of this kind of CSR is usually larger than the lumped inductor with package of 0201 and 01005. So that, the lumped inductor with package of 0201 is used in this work to move the resonant frequency of a single layer square loops FSS to lower frequency b, which can be explained by Eqs. (2), (3), (4).

**Figure 7:**
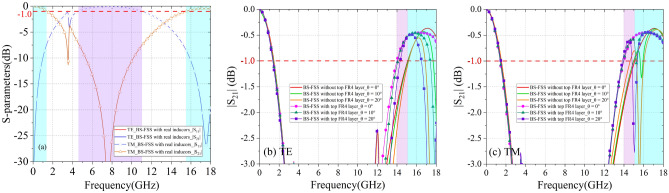
(**a**) At normal incidence, frequency response of proposed BS-FSSs. (**b**) At TE-polarization, transmission coefficients of BS-FSS with/without top FR4 layer. (**c**) At TM-polarization, transmission coefficients of BS-FSS with/without top FR4 layer.

Figure 6 illustrates the BS-FSS for proposed HADTR. The whole structure consists of two different FSSs on both sides of the FR4 substrate with ε_r_ of 4.4, tanσ of 0.025 (h_5_ = 0.3 mm), whose unit cells are different square loops loaded with distinctive chip inductors. Different from the ideal inductors, the reactance value of L cannot accurately depict the response characteristics of chip inductors. Therefore, in full-wave simulation software CST, we input the S-parameters in the range of 0.05–20.00 GHz of LQP03TG0N1B02 and LQP03TG0N4B02 chip inductors with 0201 package to ensure the accuracy of simulation results. The simulation results of transmission/reflection coefficients of band-stop FSS, band-stop FSS Top and band-stop FSS Bottom at normal incidence are shown in Fig. 7a. The parameters of band-stop FSS Top are P = 22.0 mm, *l*_1_ = 10.4 mm, w_1_ = 1.7 mm; the parameters of band-stop FSS Bottom are P = 22.0 mm, l_2_ = 9.0 mm, w_2_ = 1.1 mm. For the whole structure of BS-FSS, the transparent windows (|S_21_|≥ − 1.0 dB) are in the range of 0–1.42 GHz and 15.48–18.68 GHz; the − 1 dB reflection band is in the range of 4.62–11.02 GHz. Figure 7b–c shows transmission coefficients of BS-FSS with/without top FR4 layer. The added top FR4 layer h_1_ broadens the transmission band efficiently, while it can enforce absorptivity and stability of oblique incidence at the same time.

**Figure 8:**
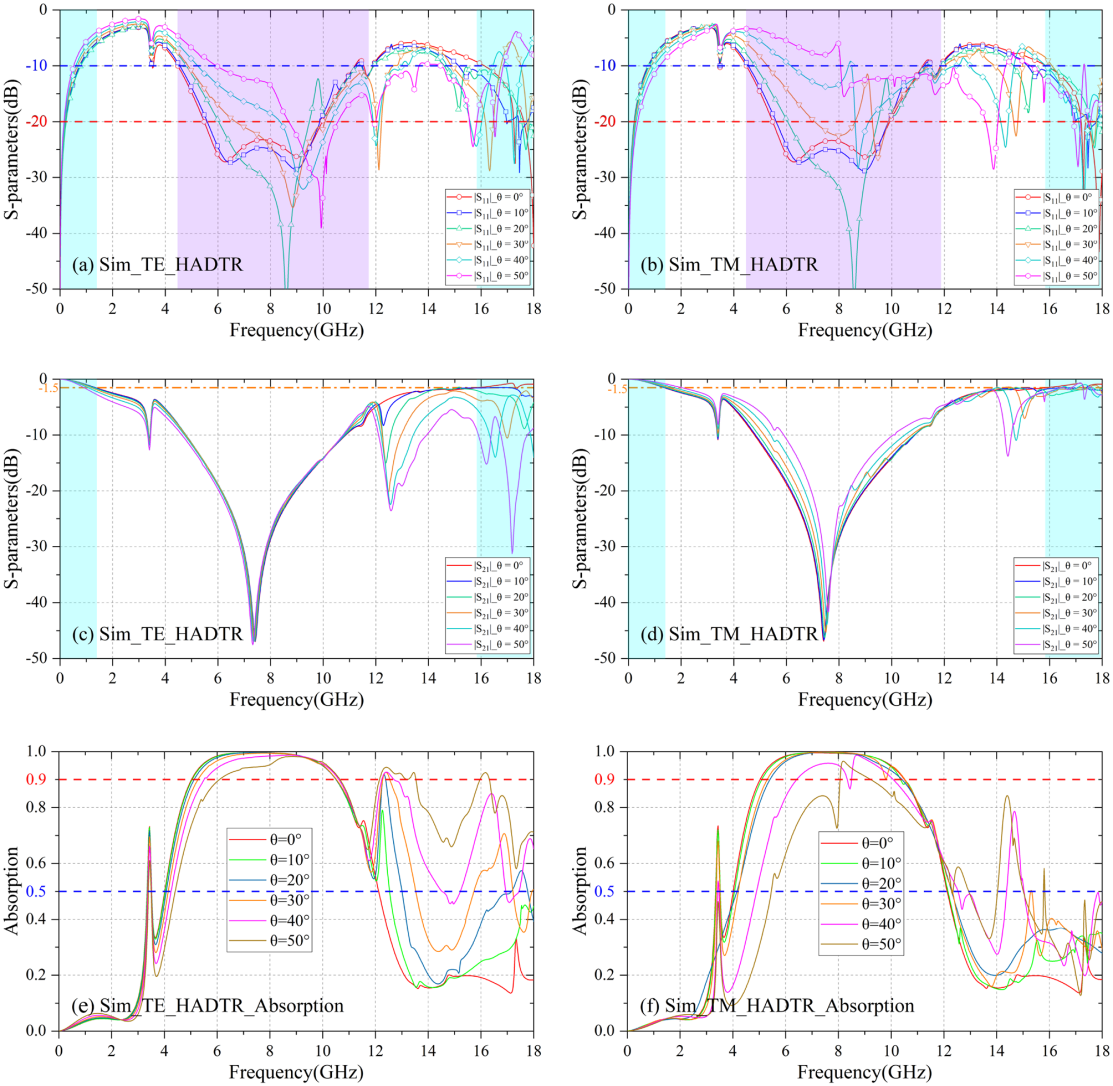
Simulation results the proposed HADTR. (**a**,**b**) At TE/TM polarization, reflection coefficients under different oblique incidences. (**c**,**d**) At TE/TM polarization, transmission coefficients under different oblique incidences. (**e**,**f**) At TE/TM polarization, absorptivity under different oblique incidences.

By replacing the metal ground of CA absorber with the BS-FSS above, the proposed HADTR is shown in Fig. 1. The simulation results of transmission/reflection coefficients and absorptivity of HADTR are shown in Fig. 8. At normal incidence, both TE and TM polarization simulation results demonstrate that the reflection coefficient of the HADTR below − 10 dB is in the range of 4.48 to 11.70 GHz (FBW = 89.2%); the reflection coefficient below − 20 dB covers a bandwidth in the range of 5.48 to 9.96 GHz (FBW = 58.0%); and the 90% absorption band is in the range of 5.02–10.56 GHz (FBW = 71.1%). The low frequency transmission band with insertion loss 1.5 dB of HADTR is up to 1.34 GHz; and the high frequency transmission band with 1.5 dB insertion loss is from 16.04 to 18.70 GHz. And the proposed HADTR possesses improved oblique incidence property. In the case of TE polarization, the fractional bandwidth of − 10 dB and 20 dB reflection is 85.1% (θ = 50°) and 58.0% (θ = 30°); when the incidence angle is 40°, FBW of 90% absorption is 62.2%; when the incidence angle is 30°, the low frequency transparent window (|S_21_|≥ − 1.5 dB) is in the range of 0–1.18 GHz. Under TM polarization, HADTR shows better oblique incidence performance than the property of TE polarization; as the oblique incidence angle increasing from 0° to 50° with a step of 10°, the low frequency transmission band (|S_21_|≥ − 1.5 dB) is extended from 0–1.34 GHz to 0–2.0 GHz; and when the incidence angle is 50°, the high frequency transparent window (|S_21_|≥ − 1.5 dB) is 16.44–17.26 GHz.

### Fabrication and measurement results

**Figure 9:**
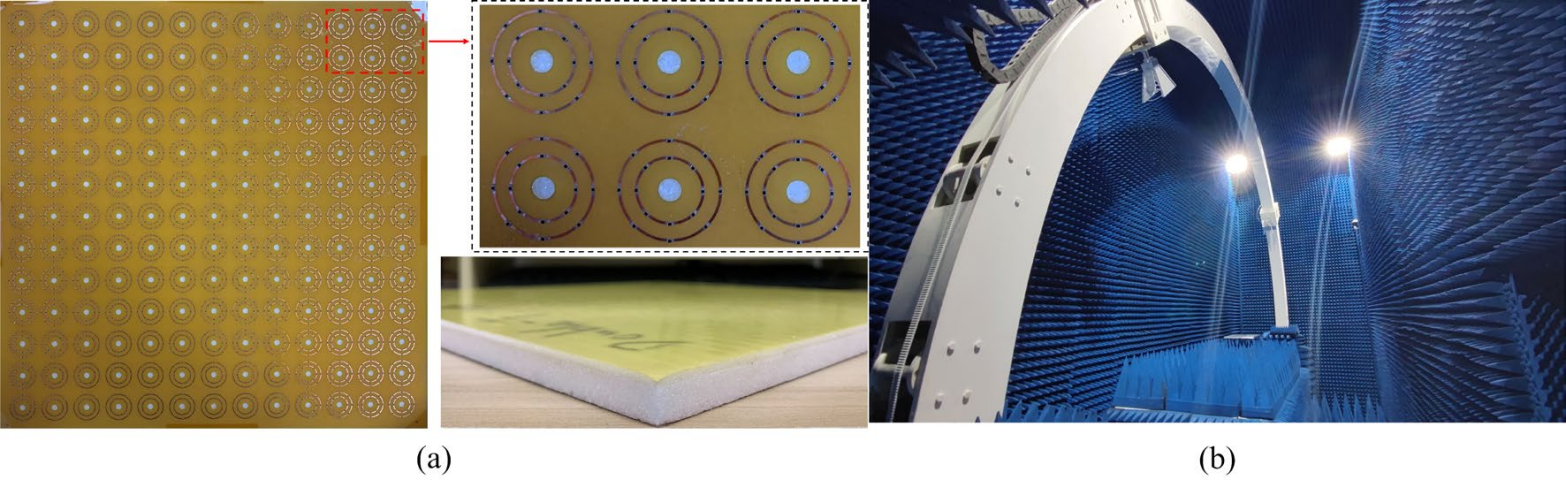
Photograph of the fabricated CA absorber prototype. (**a**) Top view of the fabricated CA sheet, Specific view of unit cell, Perspective view of the fabricated CA absorber prototype. (**b**) Measurement setup for |S_11_|.

To validate the reflection coefficient/absorptivity of the CA absorber, a prototype with same CA sheet is manufactured, which consist of 13 × 13 cells with the size of 300 × 300 mm. The CA sheet is manufactured by printing the periodic resistive FSS on a piece of PI film with thickness of 0.13 mm, ε_r_ of 3.5, tanσ of 0.0027. The prototype combines a metallic ground, a foam layer with thickness of 8.50 mm, ε_r_ of 1.05, tanσ of 0.0029, a CA sheet, a foam layer and a FR4 layer, which is adhered by four pieces of hot melt adhesive films with ε_r_ of 2.93. Figure 9a provides the specific sight of the unit cell, and the chip-resistor is with 0201 package. Figure 9b shows measurement setup.

**Figure 10:**
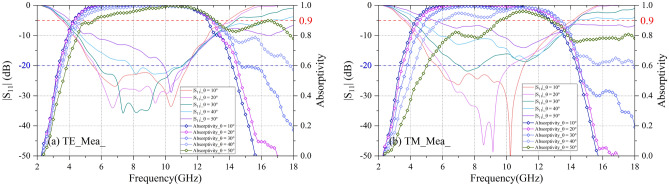
Measurement results of reflection coefficients and absorptivity under the different oblique incidences. (**a**) TE-polarization and (**b**) TM-polarization.

The experimental results of the tested prototype are plotted in Fig. 10. Under TE-polarization, for 10° angle of oblique incidence, − 10 dB and − 20 dB absorption band of Prototype is 4.27–12.64 GHz (FBW = 114.4%) and 5.47–11.42 GHz (FBW = 70.5%); for 30° angle of oblique incidence, − 20 dB absorption band is reduced slightly to the range of 5.81–11.05 GHz (FBW = 62.2%); for 40° angle of oblique incidence, − 10 dB absorption band is in the range of 4.74–13.05 GHz (FBW = 96.5%). Under TM-polarization, for 10° angle of oblique incidence, − 10 dB and − 20 dB absorption is 4.33–12.69 GHz (FBW = 114.4%) and 5.66–11.06 GHz (FBW = 64.6%) respectively; for 30° angle of oblique incidence, − 10 dB absorption is from 4.96 to 13.35 GHz (FBW = 91.6%). The solid agreement between the simulation and measurement validates design idea of the proposed CA absorber.

**Figure 11:**
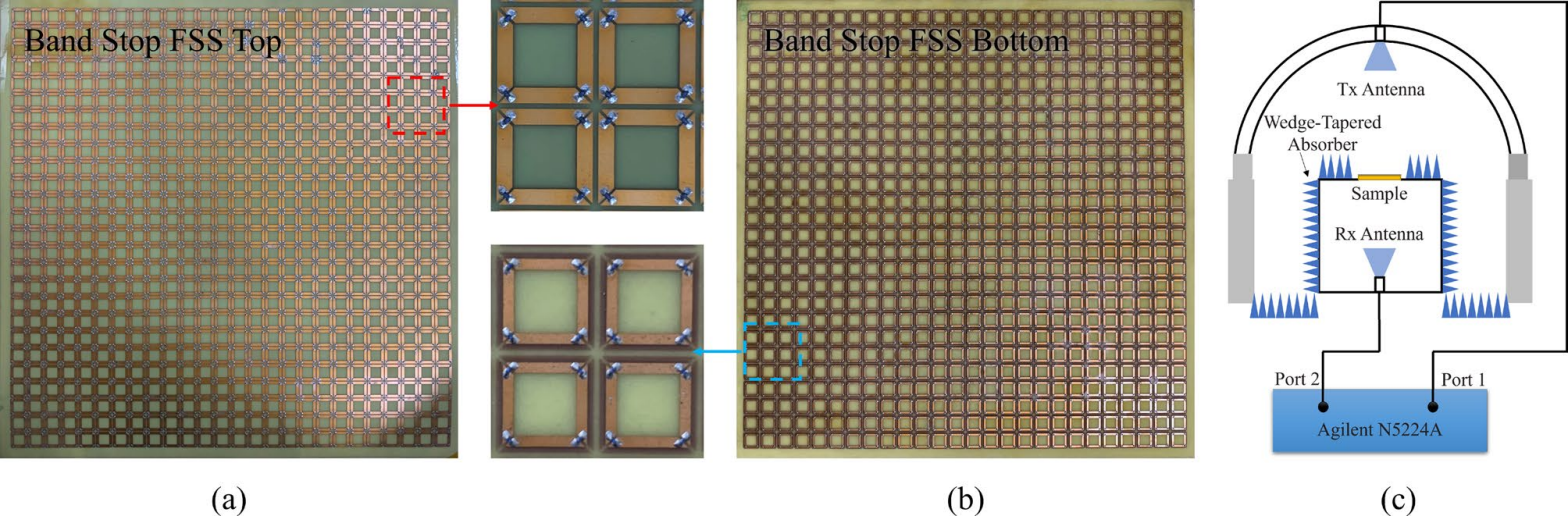
Photograph of the fabricated BS-FSS prototype. (**a**) Top side of the BS-FSS. (**b**) Bottom side of the BS-FSS. (**c**) Measurement setup for |S_21_|.

**Figure 12:**
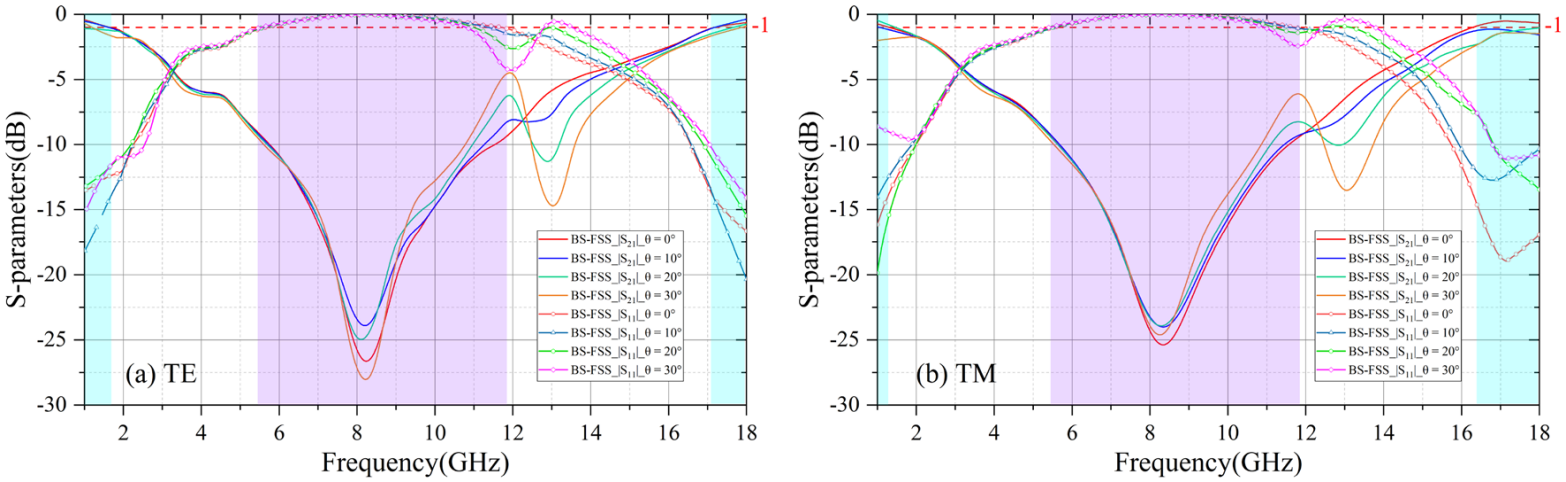
Measurement results of the proposed BS-FSS Prototype: (**a**) At TE-polarization, reflection/transmission coefficients under different oblique incidences. (**b**) At TM-polarization, reflection/transmission coefficients under different oblique incidences.

Figure 11 shows the sample of BS-FSS, which consists of 26 × 26 cells with the size of 300 × 300 mm. And the thickness of the FR4 substrate is h_5_ = 0.3 mm. The series numbers of chip inductors L_1_ and L_2_ are LQP03TG0N1B02 and LQP03TG0N4B02 with value 0.1 nH and 0.4 nH respectively. Figure 12a,b shows the experimental results of the samples within incidence of 30°. At normal incidence, under TE polarization, − 1 dB reflection band is in the range of 5.54–11.66 GHz. The low frequency transmission band with 1 dB insertion loss is in the range of 0–1.53 GHz. The high frequency transmission transparent window with 1 dB insertion loss is in the range of 16.91–18.00 GHz. At normal incidence, under TM polarization, − 1 dB reflection band is in the range of 5.56–11.73 GHz. The low frequency transmission band with 1 dB insertion loss is in the range of 0–1.31 GHz. The high frequency transmission transparent window with 1 dB insertion loss is in the range of 16.28–18.00 GHz. It should be noticed that the proposed BSS-FSS shows stable reflection coefficient when the oblique incidence is less than 30° under both TE and TM polarization.

**Figure 13:**
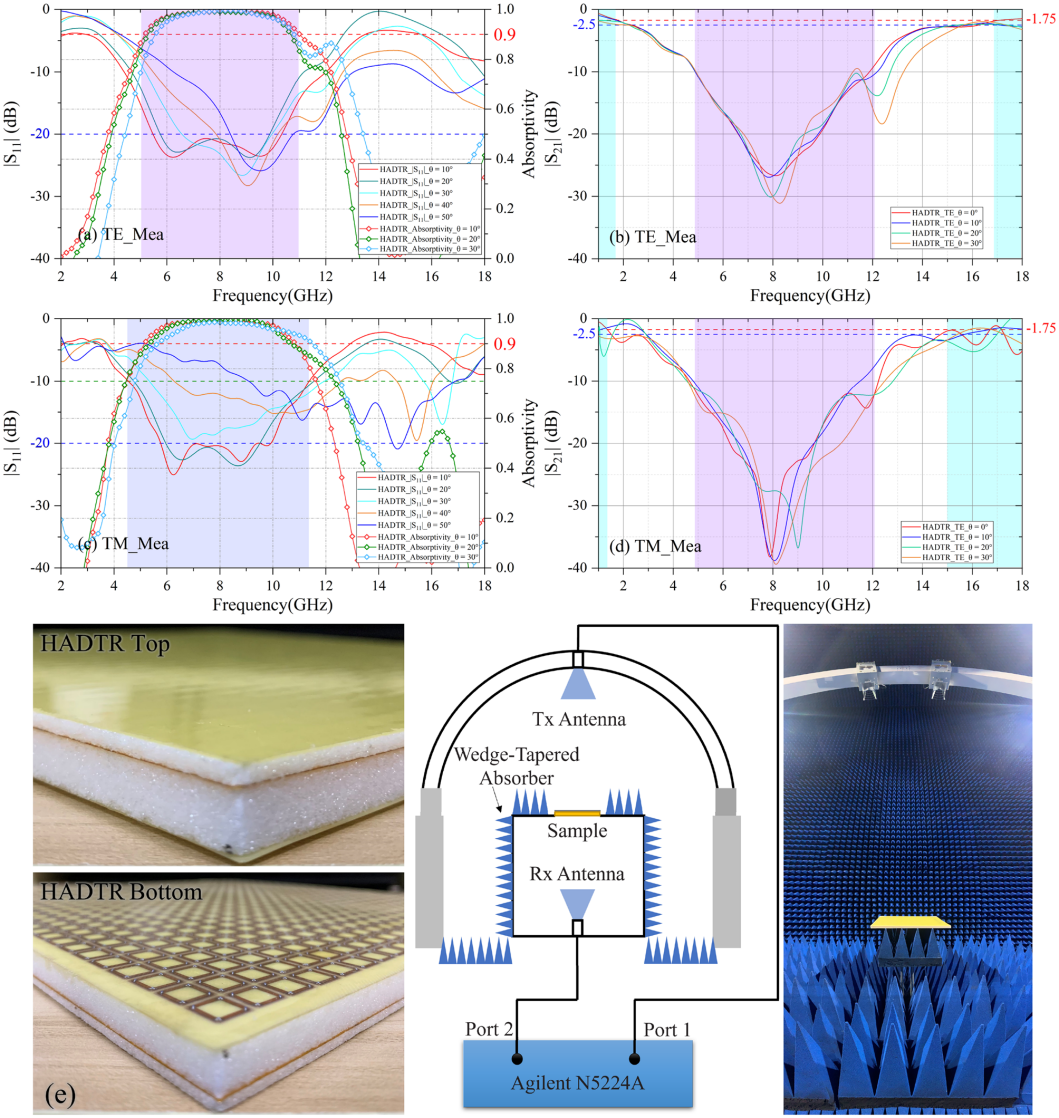
Measurement results the proposed HADTR. (**a**) At TE-polarization, reflection coefficients and absorptivity under different oblique incidences. (**b**) At TE-polarization, transmission coefficients under different oblique incidences. (**c**) At TM-polarization, reflection coefficients and absorptivity under different oblique incidences. (**d**) At TM-polarization, transmission coefficients under different oblique incidences. (**e)** Photograph of the fabricated HADTR prototype and Measurement setup for |S_21_|/|S_11_|.

By combining the CA absorber Prototype and BS-FSS prototype, the sample of the proposed HADTR is obtained. Figure 13e shows the photograph of the fabricated HADTR and measurement setup for |S_21_| and |S_11_|. Figure 13a,c shows measurement results of reflection coefficients and absorptivity of the HADTR prototype. When the incidence angle is 10°, in the case of TE-polarization, − 10 dB reflection band of the HADTR is in the range of 4.45–12.06 GHz (FBW = 92.2%); − 20 dB reflection band covers a bandwidth in the range of 5.44–10.38 GHz (FBW = 62.5%); and the 90% absorption band is in the range of 5.08–11.03 GHz (FBW = 73.9%); in the case of TM-polarization, − 10 dB reflection band of the HADTR is 4.55–11.37 GHz (FBW = 85.7%); − 20 dB reflection band covers a bandwidth in the range of 5.77–9.92 GHz (FBW = 52.9%).

Figure 13b shows the measurement results of transmission coefficients at TE-polarization. At normal incidence, the low frequency transmission band with insertion loss 1.5 dB of HADTR is up to 1.42 GHz; and the high frequency transmission band with 2 dB insertion loss is from 16.71 to 18.00 GHz. As shown in Fig. 13d, at TM polarization, when the incidence angle in less than 20°, there are always two transmission bands in the range of 1.00- 2.00 GHz and 15.00–18.00 GHz. Compared with simulation results, we think the increased insertion loss is caused by the PMI foam slabs of the structure and hot melt adhesive film, which can be improved by using other dielectric material and adhesive film. With increasing of oblique incidence angles, the measurement results for both TE and TM polarization demonstrate the same tendency of simulation results.

Table 1 lists the performance comparisons between the proposed HADTR and others in the literature. Though the − 10 dB reflection band of some designs are larger than the proposed HADTR, they show the characteristic of sensitive oblique incidence performance. At the same time, the range of their − 20 dB reflection bands is limited, but the proposed HADTR has wide − 20 dB reflection band when the oblique incidence angle is lower than 30°. And by comparing FBW of 90% absorption, it can be seen that HADTR is the only design which has ultrawide bandwidth with advantage of stable oblique incidence performance. Meanwhile, it should be noticed that 90% absorption bands of these compared designs are much narrower than their − 10 dB reflection bands.

**Table 1:** Performance comparison of TAT rasorbers, relative thickness (RT), which corresponds to the starting frequency of − 10 dB reflection (λ_L_).

References	Abs. band FBW/oblique			Tran. band (GHz)	RT λ_L_
	− 10 dB reflection	− 20 dB reflection	90% Absorption	− 1.5 dB Insert loss	
^26^	100%/–	Narrow peaks	57.9%/–, 36.8%/–	7.47–7.93, 12.47–12.72	0.099
^28^	129.4%/20°	Narrow peaks	36.4%/30°, 19.8%/20° 12.0%/0°	8.08–8.47, 12.69–13.31	0.078
^29^	43.6%/-, 65.5%/-	Narrow peaks	N/A	8.00–9.12, 10.33–11.63	0.091
^30^	74.4%/30°	16.9%/20°	N/A	8.34–8.86, 11.59–12.47	0.117
^31^	111.4%/30°	Narrow peaks	15.8%/30°	5.86–6.86, 9.68–11.04	0.097
This work	85.7%/50°	58.0%/30°	71.3%/50°	0–1.34, 16.04–18.00	0.171

## Discussion

This work has proposed a HADTR with high absorption and improved oblique incidence performance. High absorption and improved oblique incidence performance can be ascribed to its composition unit cell with absorption enforced design, which CA sheet for HADTR is obtained by absorptivity analysis and avoids the complicated process of extracting RCL from equivalent circuit model; then PMI and FR4 superstrate is added on the top of resistive layer to improve absorption further. To design a BS-FSS with a high reflection and dual-transmission bands, double metallic square loops on both sides of FR4 substrate are loaded with chip inductors, which exhibits stable reflection coefficient when the oblique incidence is less than 30°. A prototype HADTR is fabricated and measured, and the measured results are in good agreement with the simulated results. The work demonstrates that the FSRs can achieve excellent performance by simple unit cells, which may create a new way and help researchers efficiently design future-generation rasorbers.

## Methods

### Measurement

As shown in Figs. 9b and 13c, the samples were measured by an Agilent N5224A vector network analyzer (VNA). One standard-gain horn antenna was used as transmitting antennas; another standard-gain horn antennas was used as receiving antennas for reflected signal; the third standard-gain horn antenna was used as receiving antennas for transmitted signal. Each antenna was connected to the VNA. The radius of the orbit is 2.50 m. To prevent unwanted signals, the fabricated samples were surrounded by a wedge-tapered absorber. Besides, a time gating function of the vector network analyzer was employed to measure the signals caused by samples. To measure reflection coefficient, firstly, two antennas were placed at symmetrical positions about the perpendicular bisector of the prototype to allow them to radiate vertical-polarized EM waves. Then, the reflection coefficient of an aluminum alloy plate was measured and set the magnitude of its reflection coefficient to be 1 for the calibration process. Finally, the reflection coefficient of the sample can be measured accurately. To measure transmission coefficient, the sample are placed between two horn antennas which were connected to different ports of VNA aiming at the center of the sample. The transmissions between the two antennas with/without the sample were both measured and the difference between them regarded as the transmission coefficient of the prototype. As shown in Fig. 9b, the E-field of the radiated wave was parallel to the sample surface, so the oblique incident wave in this setup was TE-polarized. To measure the performance under TM-polarized oblique incidence, two antennas should be rotated by 90° so that the H-field of the radiated wave was parallel to the surface of the sample; other measurement process was the same.

### Simulation

The proposed HADTR was designed and simulated by the commercial finite integration technique (FIT) based software CST Microwave Studio (CST Computer Simulation Technology). Periodic boundary conditions were applied in the both of x and y directions, with open boundaries in the z-direction.

## Data Availability

The data that support the findings of this study are available from the corresponding author upon reasonable request.
